# Mobile SARS‑CoV‑2 screening facilities for rapid deployment and university‐based diagnostic laboratory

**DOI:** 10.1002/elsc.202200026

**Published:** 2023-01-03

**Authors:** Nils Stanislawski, Ferdinand Lange, Christian Fahnemann, Christoph Riggers, Marc‐Nils Wahalla, Marc Porr, Fabian Cholewa, Rebecca Jonczyk, Stefanie Thoms, Martin Witt, Frank Stahl, Sascha Beutel, Andreas Winkel, Philipp‐Cornelius Pott, Meike Stiesch, Mira Paulsen, Anette Melk, Henning Lucas, Stefanie Heiden, Holger Blume, Cornelia Blume

**Affiliations:** ^1^ Institute of Microelectronic Systems Architectures and Systems Group Leibniz University Hannover Hannover Germany; ^2^ Institute of Technical Chemistry Leibniz University Hannover Hannover Germany; ^3^ Department of Prosthetic Dentistry and Biomedical Materials Science Hannover Medical School Hannover Germany; ^4^ Department of Pediatric Kidney Liver, and Metabolic Diseases Hannover Medical School Hannover Germany; ^5^ Institute of Innovation Research Technology Management and Entrepreneurship Leibniz University Hannover Hannover Germany; ^6^ Lower Saxony Centre for Biomedical Engineering Implant Research and Development (NIFE) Hannover Germany

**Keywords:** COVID‐19, HIS & LIMS, PCR, SARS‐CoV‐2, screening

## Abstract

The severe acute respiratory syndrome coronavirus 2 (SARS‐CoV‐2) pandemic has created a public crisis. Many medical and public institutions and businesses went into isolation in response to the pandemic. Because SARS‐CoV‐2 can spread irrespective of a patient's course of disease, these institutions’ continued operation or reopening based on the assessment and control of virus spread can be supported by targeted population screening. For this purpose, virus testing in the form of polymerase chain reaction (PCR) analysis and antibody detection in blood can be central. Mobile SARS‐CoV‐2 screening facilities with a built‐in biosafety level (BSL)‐2 laboratory were set up to allow the testing offer to be brought close to the subject group's workplace. University staff members, their expertise, and already available equipment were used to implement and operate the screening facilities and a certified diagnostic laboratory. This operation also included specimen collection, transport, PCR and antibody analysis, and informing subjects as well as public health departments. Screening facilities were established at different locations such as educational institutions, nursing homes, and companies providing critical supply chains for health care. Less than 4 weeks after the first imposed lockdown in Germany, a first mobile testing station was established featuring a build‐in laboratory with two similar stations commencing operation until June 2020. During the 15‐month project period, approximately 33,000 PCR tests and close to 7000 antibody detection tests were collected and analyzed. The presented approach describes the required procedures that enabled the screening facilities and laboratories to collect and process several hundred specimens each day under difficult conditions. This report can assist others in establishing similar setups for pandemic scenarios.

**Abbreviations**
BSL: biosafety level
HA: high availability
HIS: health information system
LIMS: laboratory information management system
PCR: polymerase chain reaction
QR: quick response
RKI: Robert Koch‐Institute
RNA: ribonucleic acid
RT‐qPCR: reverse transcription‐quantitative PCR
SID: specimen identifier
SOP: standard operating procedure
VM: virtual machine
VPN: virtual private network

## 1 INTRODUCTION

The first year of the severe acute respiratory syndrome coronavirus 2 (SARS‐CoV‐2) pandemic was characterized by serious long‐term impacts on society and the economy. The rapid spread of SARS‐CoV‐2 is intensified by asymptomatic carriers and pre‐symptomatic transmission [2, 3]. Medical institutions such as hospitals and nursing homes rapidly limited access for visitors and put strict hygiene directives into place. The pandemic also drastically impacted educational institutions. Classroom teaching shifted to distance learning, and research activity in universities was restricted to curb the spread of SARS‐CoV‐2. Many businesses went into isolation, significantly affecting critical supply chains and small enterprises without sufficient funds for long non‐working periods. Public health measures often mitigate spread through diagnostic testing, vaccination, and self‐isolation. Population screening to identify infectious individuals in combination with other control efforts constitutes one approach of breaking transmission chains to suppress the ongoing pandemic and reopen societies [4, 5].

Population screening has to provide sufficient accessibility and throughput capabilities, resulting in a high test frequency (i.e., less than the viral incubation period) with fast sample‐to‐answer times in order to represent effective measure for the assessment and control of virus spread [4]. Various testing modalities were used worldwide to contain the pandemic. Drive‐through testing centers, home visiting testing, and walk‐through centers represent the most common approaches, with some groups also providing mobile testing facilities [6, 7, 8, 9, 10, 11, 12, 13]. Since the beginning of the SARS‐CoV‐2 pandemic, polymerase chain reaction (PCR) tests have routinely been used to confirm an acute infection and are still considered the gold standard for diagnostic tests. As of spring 2021, rapid antigen tests have become a primary frontline tool in detecting SARS‐CoV‐2 in the general public because they outperform PCR tests regarding the analysis period and cost, and can often be performed by subjects themselves. In contrast, rapid antigen tests lack the analytical sensitivity of PCR tests, especially in asymptomatic subjects [14, 15]. PCR tests are still used to confirm an acute infection, even after a positive rapid antigen test. However, PCR testing capacity in Germany remains constrained by supply chain shortages and the limited bandwidth of diagnostic labs [16].

Many university research laboratories provided SARS‐CoV‐2 molecular diagnostic testing in response to the pandemic and test capacity shortage [8, 11–13]. However, most universities, especially those without a medical school, lacked the capacity of clinical diagnostic labs at the beginning of the pandemic. Container‐based laboratory setups have proven suitable in practice for the detection and containment of infectious diseases due to their portability when focusing on potential sources of infection [17, 18]. Setting up a diagnostic laboratory takes an immense amount of work, particularly considering the diagnostic, safety, security, supply chain, logistics, digital infrastructure, legal, and financial requirements.

The project described in this article was intended to create additional testing capacity using university facilities, staff members, and their expertise. The project was conducted at a university without a medical school, but was supported by individual institutions of a medical university in planning, organization, and medical guidance. At the outset of the project, a sufficient number of biosafety level (BSL)‐2 laboratories for the ribonucleic acid (RNA) isolation upfront of a PCR analysis were not available on campus. Mobile screening facilities for both specimen collection close to the subjects’ workplace and RNA isolation in an integrated BSL‐2 laboratory were developed to address this issue. The pre‐processed, noninfectious samples were distributed among the university's labs for PCR analysis. A custom health information system (HIS) and laboratory information management system (LIMS) were developed to support the process flow of collecting subject data and tracking and reporting samples as well as analysis results to local public health departments and subjects. The utilized screening facilities, diagnostic methods, HIS, and LIMS underwent constant development toward a more automated workflow during the project. This development includes the addition of test centers for antibody screening indicating a subject's previous SARS‑CoV‑2 infection and its immune response to infection and vaccination. The implementation of serological testing was undertaken to provide insights into the seroconversion of subjects and record the number of subclinical cases of SARS‐CoV‐2 infections. All proceedings were subject to extensive compliance with regulatory directives, mainly including certification of the diagnostic laboratory and fulfillment of health‐related data policies.

## 2 MATERIALS AND METHODS

In order to ensure timely implementation and start of operation of the screening facilities and diagnostic laboratory, the various tasks were divided among specialized working groups and university institutions. Figure 1 depicts the processes associated with provisioning consumables, collecting specimen and subject information, analysis, and reporting results to subjects and health authorities. The representation of material and data flow is shown in Figure 1A; Figure 1B shows the implementation of the laboratory and digital infrastructure processes.

FIGURE 1Processes displayed by material and data workflow. (A): Representation of material and data flow. Prepared test‐kits for PCR and antibody testing (a) were provided to screening facilities, where subject data was entered and samples (oropharyngeal swabs or blood samples) were collected (b). Swabs were handed to a BSL‐2 laboratory for RNA isolation (c), followed by PCR analysis (d). Serum samples were used for antibody detection analysis (e). Lab processes were controlled by the LIMS, which also stored raw analysis data (f). Raw analysis data was evaluated and confirmed results (g) were sent to the HIS, upon which sample result and subject data were merged (h). Positive PCR test results were submitted to the health department and a medical staff member informed the subject, whereas all other test results were sent to the subject via e‐mail (i). (B): Implementation of the laboratory and digital infrastructure processes. Subject and sample admissions started with manually entering or recalling a subject's personal information (e.g., name, address) from a previously handed‐out QR code. Subsequently, the acquisition of voluntarily given answers to medical study questionnaires in the HIS data entry software occurred. Subjects were then given printed handouts with their QR code for future visits and a consent form to be signed before taking either an oropharyngeal swab or a blood sample. The collected sample was pseudonymized with an identifier label from a pre‐assembled test kit, and the completed dataset was stored in the HIS database. Depending on the type of specimen, samples were then handed to the appropriate laboratory for analysis. Upon retrieval, the arrival of samples was documented. For PCR and antibody analysis, plate and rack information were created by scanning and sorting samples. Analysis results were evaluated and confirmed by lab managers. If no valid result was achieved and a reserve sample was available, analysis was performed again. Confirmed results (including the need for sample recollection) for each pseudonymized sample were sent to the HIS database for report generation and informing subjects. In case of a positive PCR test result, a medical staff member received the subject and sample information to contact the subject directly. At the same time, the report was sent to the public health department

**Figure:**
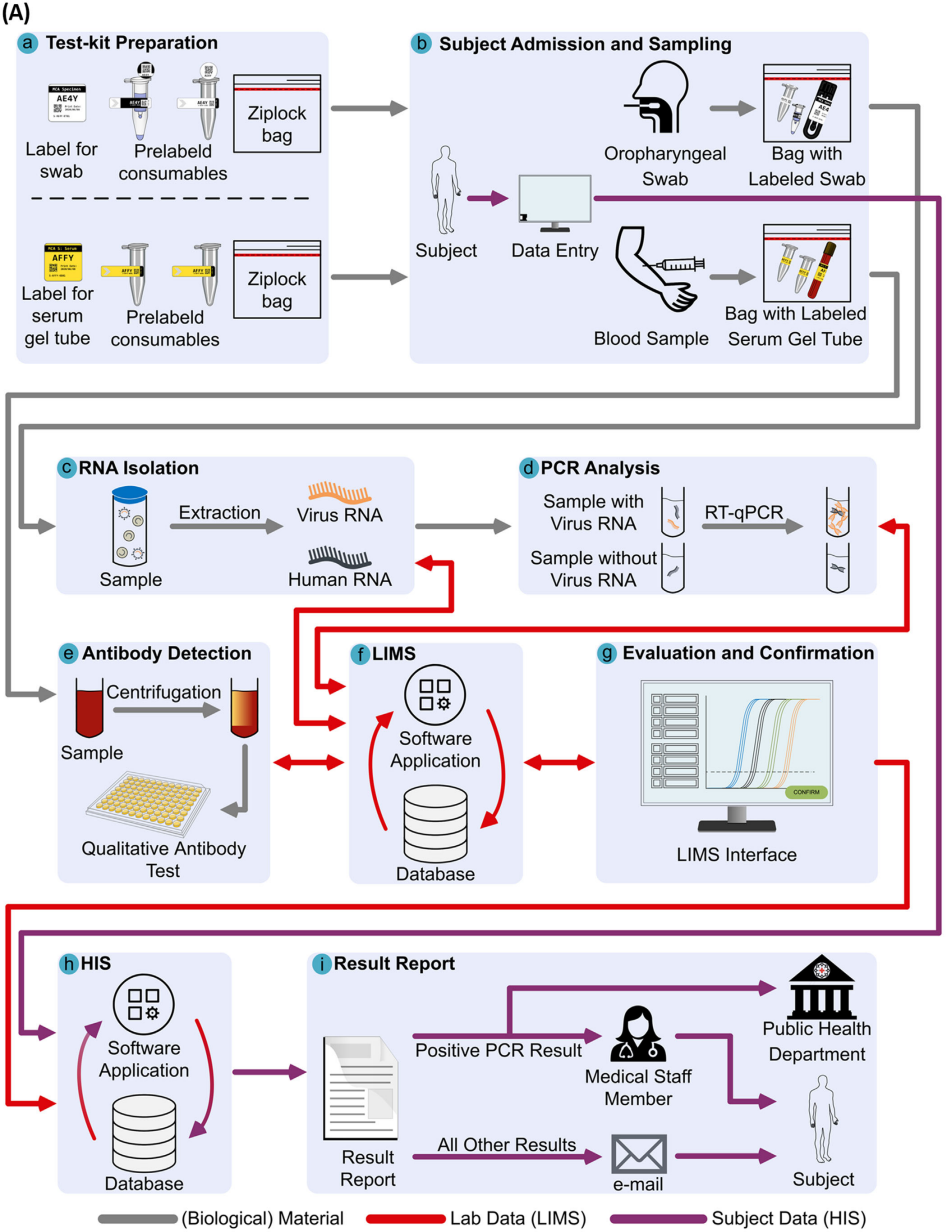

**Figure:**
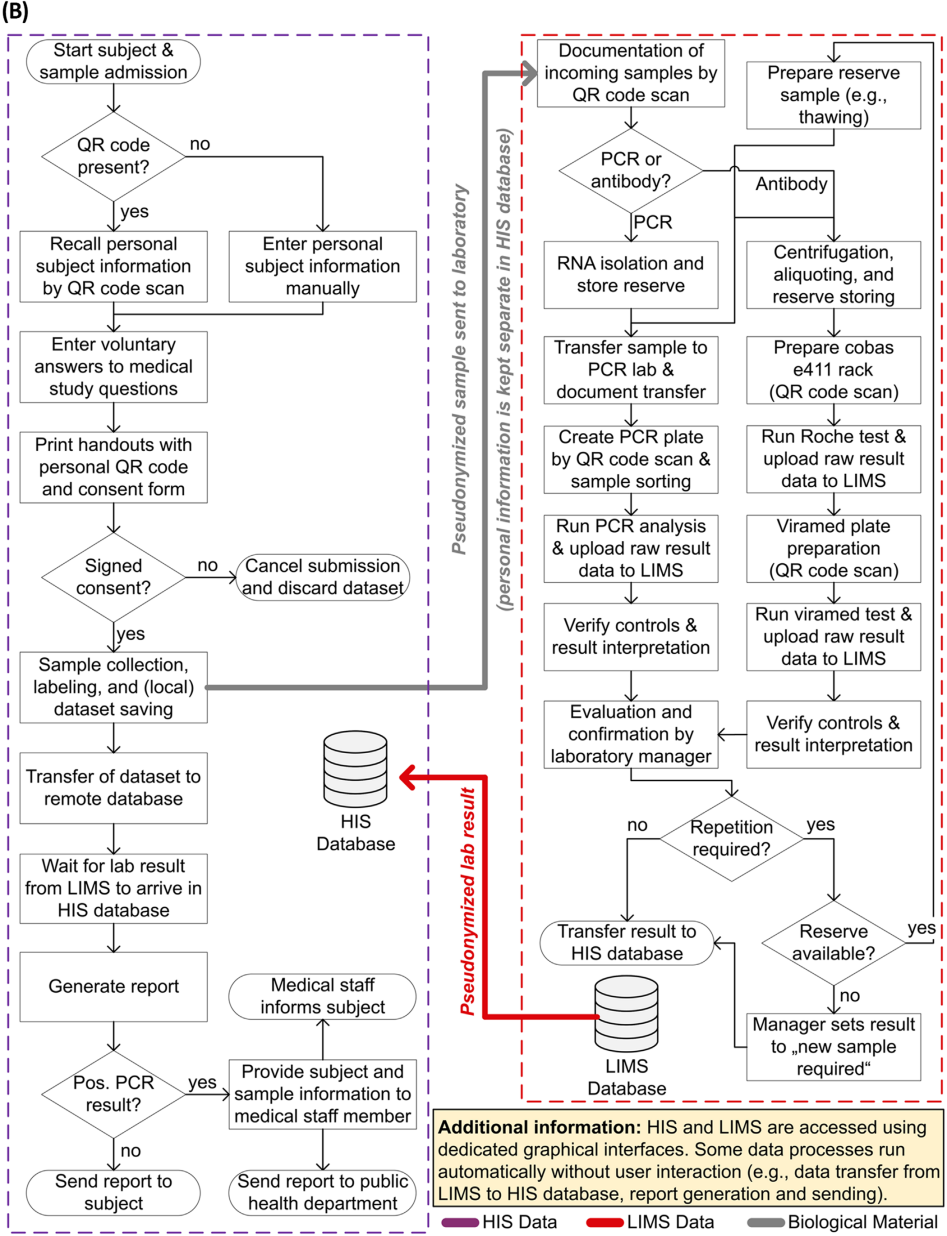

**PRACTICAL APPLICATION**

Population screening for SARS‐CoV‐2 presents one possible means to control infection spreading. PCR tests remain the gold standard for the detection of an acute SARS‐CoV‐2 infection. University‐based diagnostic laboratories can provide much required PCR testing capacities due to their expertise and equipment. This article summarizes and shares how screening facilities and diagnostic laboratories can be set up and operated under the difficult conditions (e.g., supply chain shortages) of a pandemic. An important feature of the screening facilities described is their mobility, which allows them to be set up flexibly close to groups of test subjects. The described integration of a BSL‐2 laboratory into a container‐based screening facility enables the expansion of laboratory capacities. The modular description of the laboratory methods used and the setup of the (digital) infrastructure enables adaptation based on existing expertise and equipment. The software and documentation developed as part of this work are publicly available online to assist others in setting up further studies [1].

### 2.1 Operational coordination and logistics

Main challenges—especially during the initial phase of the SARS‐CoV‐2 pandemic—included the supply and provisioning of consumables and safety equipment. These tasks were therefore implemented and maintained in‐house. This mainly included the in‐house production of test kits and disinfectants after a change in the corresponding legal basis [19]. Transport of samples and waste was handled according to transnational transport of hazardous materials (i.e., ADR) regulations, which were supplemented by special regulations within the framework of national measures due to the pandemic [20]. Overall, about 150 persons were engaged in collecting samples, performing laboratory analyses, providing material, coordinating staff and information, and the continuous developing of procedures. A large proportion of staff members comprised students and PhD candidates enrolled in life sciences, engineering, and medical courses. Daily self‐assessment of medical and laboratory personnel ensured detection of acute infections as early as possible to prevent transmission within the team.

To uniquely identify specimens, custom adhesive labels were provisioned. Each label contains a unique specimen identifier (SID) in both a human‐readable and a machine‐readable quick response code (QR code) format for automatic data entry using a handheld scanner. The SIDs are defined as unsigned integers encoded using a character set based on the RFC3548 specification [21]. Only unambiguous capital letters and numbers are used in this encoding, eliminating the need to differentiate between similar‐looking characters (i.e., O and 0, I and 1). The SID features an additional symbol for error detection of common human mistakes like mistyping or swapping of characters. This ensures error‐free manual data input in case of scanner faults. The SID is extended with a suffix for the declaration of specimen type and the specifics of consumable material. This enabled software‐driven quality control of each prepared test‐kit.

Test kits with prelabeled consumables specific to the performed analysis were prepared in‐house. PCR test kits consisted of labeled reaction tubes and RNA columns placed in clear zip bags; test kits for antibody detection only contained labeled reaction tubes. Each test kit also included additional adhesive labels for uniquely identifying swab and serum gel tubes directly after sample collection. Swab tubes and serum gel tubes were not included in the prepackaged test kits and instead provided in bulk packages at the testing location to account for repeated sample collection in case of initial failure. The correct assembly of test kits was recorded in a standard operating procedure (SOP) and each assembled kit was digitally verified by scanning its content. To ensure RNase‐free assembly, all work areas used were cleaned and disinfected prior to test kit packaging, including RNaseZap (Sigma–Aldrich; St. Louis, Missouri).

### 2.2 Subject selection and specimen collection

The targeted population groups for PCR screening were selected regarding an increased infection risk based on professional activity and continuity of the provided medical care structure. In this regard, employees of medical workplaces (especially nursing homes), educational institutions, and companies of systemic importance were offered participation in the PCR test offer. Invitations were specially extended to subjects meeting the following guidelines recommended by the Robert Koch‐Institute (RKI): being over 50 years of age, having comorbidities (e.g., diabetes, obesity, cardiac, pulmonary, circulatory, or immunocompromising diseases), or taking immunosuppressants  [22]. Individuals in non‐medical occupational groups were preferentially considered to be particularly exposed if they had many contacts with people during the corona crisis due to their occupational activities (working in service areas, frequent customer contact, or abundant contact with colleagues). The PCR testing offer focused on asymptomatic subjects; however, individuals with acute symptoms possibly characteristic for COVID‐19 on the day of testing were allowed to participate in the PCR screening. Persons, who received the offer to be tested, could anonymously refuse to participate without giving a reason. PCR testing started in April 2020 with an initial container‐based screening facility set up on a university campus. Two similar screening facilities were deployed at a supplier of pharmaceutical and laboratory equipment and a school within 2 months. For subjects at different schools and nursing and retirement homes, on‐site facilities were provided with the necessary equipment for subject data entry and sample collection. Antibody screening tests started in August 2020 and were offered to all groups already participating in the PCR test offer.

In times of high SARS‐CoV‐2 incidence, subjects were invited for weekly participation in the PCR test offer, with biweekly tests recommended during phases of low incidence. A pharyngeal smear was performed for PCR testing by swabbing the region between the anterior and posterior palatal arches on the subject. Most throat swabs were performed at a mobile screening facility by trained medical personnel. At nursing and retirement homes, swabs for PCR testing were taken in collaboration with physicians and senior staff members and were collected on‐site and picked up for further processing. Teachers at three schools provided self‐collected pharyngeal swabs after receiving appropriate in‐person and video supported training. Subject participation in antibody detection tests was offered twice in the autumn of 2020 and spring of 2021. Blood samples for detecting SARS‐CoV‐2‐specific antibodies were taken from the cubital vein by certified medical personnel. After informed consent, samples, subjects’ contact information, and clinical parameters were collected.

Furthermore, subjects were questioned on each day of testing whether COVID‐19 symptoms or risks regarding SARS‐CoV‐2 infection were or had been present. Later, vaccination status of subjects was also inquired. These additional parameters were answered voluntarily at each appointment to support epidemiological research. During the first time being tested, subjects received a printout featuring an encrypted QR code containing the provided contact information to accelerate data entry during subsequent tests. After sample collection, the labeled sample tube (either obtained from a pharyngeal swab or blood sampling) and its respective test kit were then passed to the appropriate laboratory for analysis. By physically separating subject information and the SID at this point, only pseudonymized samples were given to the laboratory.

### 2.3 Screening facilities

**FIGURE 2:**
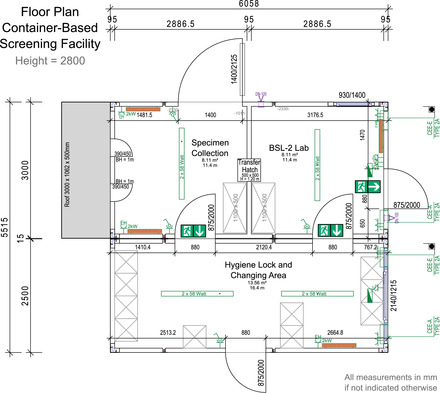
Floorplan of the container‐based screening facility. The setup consisted of two containers, of which the lower one comprised the main staff entrance, storage, server rack, and changing areas. The specimen collection as well as data entry area comprised half of the other container, with two similar working stations and windows facing outsides for subjects to line up. The second half was occupied by a BSL‐2 laboratory used for RNA isolation and was connected to the sampling area via a transfer hatch

To ensure a high acceptance and participation of the target population groups, mobile container‐based screening facilities were developed to be placed as close as possible to the targeted population while also providing the required BSL‐2 laboratories on‐premise. Each screening facility consisted of two intermodal (“shipping”) containers connected along their longitudinal side. Figure 2 displays the floor plan of the 6 m by 5.5 m setup. Manufacturing and setup of the two containers, including installation of windows, doors, and electrical wiring, were done in collaboration with FAGSI Vertriebs‐ und Vermietungs‐GmbH (Morsbach, Germany). The container setup was designed to plug into a three‐phase mains supply and run from a water tank. The deployed systems were designed to either rely on existing on‐site infrastructure or use mobile networks to establish an internet connection. The containers were transported by truck and placed on a suitably firm base (e.g., a car park). Relocation and setup of the two equipped containers took place within 1 day, and an additional day was required to install lab furniture, electronic components, and security systems.

One major challenge for container‐based laboratory setups is compliance with the appropriate BSL and ISO requirements (e.g., DIN EN ISO 15189). Following BSL‐2 specifications, all interior surfaces, including the selected technical appliances such as computers, keyboards, and handheld scanners used for sample tracking, were made out of easily sterilizable materials. To further adhere to a BSL‐2 containment level, waste was stored in designated containers placed in each section which were regularly cleared and decontaminated prior to disposal.

**FIGURE 3:**
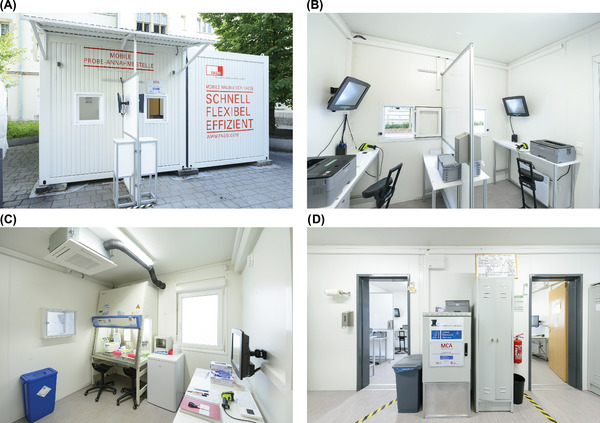
Views of the container‐based screening facility. Displayed are the two stations for subject admission and specimen collection from the outside (A) and the respective workstations inside (B). The BSL‐2 laboratory is connected to the sampling area via a transfer hatch (C). Sampling area and laboratory can be reached through the changing and storage area (D)

One container comprised the main staff entrance leading into a hygiene lock, featuring two medical and laboratory staff changing room areas. This container also stored consumables (e.g., protective clothing, swabs, etc.) and a secured server rack (see Figure 3D). The second container featured the specimen collection area and the laboratory section used for the RNA isolation. Swabs were taken at two similar working stations in the specimen collection area, designed to offer optimal protection for the medical personnel. Each of the two working stations featured a window facing outside (see Figure 3B) for subjects lining up in front. Computer screens placed close to each window allowed test persons to verify all subject‐related information entered during the specimen capture procedure. Legally‐required consent forms were printed on‐demand. These consent forms were to be signed by the subject and placed in a locked collection box in front of the window (see Figure 3A).

Collected specimens were passed to the laboratory section using a transfer hatch. This section featured specimen storage options and a class 2 safety cabinet providing two fully equipped working stations for the RNA isolation of the potentially infectious specimen (see Figure 3C). A laboratory workstation facilitated the tracking of specimens when entering or leaving the laboratory by scanning the SID.

The devised container setup remained nearly unchanged for the project duration, only with the later addition of an air‐conditioning unit to provide appropriate temperature conditions for laboratory work and storage of the utilized materials during summer. In addition to the container‐based screening facilities, reduced setups consisting only of the technical equipment required for data entry and cooled storage containers were deployed at various locations to collect oropharyngeal swabs for PCR analysis. These stations were primarily deployed at nursing and retirement homes and schools, provided that the spatial conditions available on site permitted safe operation. The collected swabs were then transported to one of the container‐based screening facilities with built‐in BSL‐2 for RNA isolation. The reduced setups were also used to collect blood samples for antibody analysis. The technical equipment required for the container‐based screening facilities and reduced setups is listed in Table 1.

**TABLE 1:** Technical devices required for the setup of the container‐based screening facilities with BSL‐2 laboratory and reduced setup stations

Setup	Location	Device	Qty.
Container	Sampling area	Data entry client (all‐in‐one personal computer)	2
		Outdoor display	2
		Disinfectable keyboard with touchpad	2
		Handheld scanner	2
		Printer	2
	BSL‐2 Lab	Safety workbench	1
		Laboratory equipment for RNA isolation (depending on used method)	1
		Refrigerator	1
		Lab client (all‐in‐one personal computer)	1
		Disinfectable keyboard	1
		Handheld scanner	1
	Changing & storage area	Cache server	1
		GSM/WiFi router	1
		UPS	1
Reduced		Data entry client	1
		Screen	2
		Disinfectable keyboard with touchpad	1
		Handheld scanner	1
		Printer	1
		Cooling transport box	1

Before the end of the deployment of a container‐based screening facility, deregistration of the BSL‐2 laboratory was filed with the responsible trade supervisory office and health department. Here, a 30‐day period had to be complied with. Technical and laboratory equipment was transferred to other facilities within the university for further use in research and educational projects after a 3‐week cooldown period. As there was no further need for mobile laboratories after the end of the project, the cleared and cleaned containers were repurposed to be used as mobile classrooms, among other things.

### 2.4 RNA isolation and PCR analysis

Initially, RNA isolation was solely performed manually using a viral RNA Mini Kit from Qiagen (QIAamp Viral RNA Mini Kit; Maryland, USA) according to the manufacturer's instruction. The swabs obtained by an oropharyngeal smear were immersed in lysis buffer. 50% of the resulting suspension of the analysis material was subjected to RNA isolation according to Chomczynski and Sacchi, leaving a reserve sample for retesting [23]. All steps required for RNA isolation were performed in the BSL‐2 laboratory section of the container‐based screening facility. The isolated RNA was used in PCR analysis.

To increase throughput, semi‐automatic RNA isolation using Qiagen's QIAcube (Qiagen; Maryland, USA) was tested during the further course of the project since the underlying RNA isolation method remained unchanged. Only slight modifications of the established SOP were required, and simultaneous isolation of 12 specimens was possible.

Later on, a predominantly automatic workflow for RNA isolation was implemented using the MagMAX Viral and Pathogen Nucleic Acid Isolation Kit by Thermo Fisher (Waltham, USA) alongside the automated Auto‐Pure 96 Nucleic Acid Purification System by Allsheng (Hangzhou, China). This workflow only requires the swab to be placed in RNA lysis buffer in the container‐based BSL‐2 laboratory. This step inactivates present RNases and stabilizes intact viral and human RNA. After this step, the specimen is no longer considered infectious and can be transferred to a BSL‐1 laboratory for automatic RNA isolation. Reformatting of the prepared lysate from reaction tubes to 96‐deepwell‐plates was performed with an epMotion 5073 Liquid Handling System by Eppendorf (Hamburg, Germany), followed by the automatic RNA isolation using the Allsheng purification system. For PCR analysis, the isolated RNA was transferred into reaction tubes using an epMotion or an rLINE Robotic Liquid Handling Dispenser Module by Sartorius (Göttingen, Germany).

Direct detection of SARS‐CoV‐2 RNA by real‐time reverse transcription‐quantitative PCR (RT‐qPCR) is considered the gold standard in diagnostics. Following the protocol described by Corman et al. and recommended by the WHO, RT‐qPCR was performed using TaqMan Fast Virus 1‐Step Master Mix by Applied Biosystems (Waltham, USA) [24, 25]. SARS‐CoV‐2‐specific PCR using a CFX384 rtPCR Detection System by Bio‐Rad (Feldkirchen, Germany) was conducted in triplicates per analyzed gene and classified as positive if two of the three results per gene were positive. The sufficiency of the pharyngeal swab was proven by a marker (human RNase P). Samples with insufficient material were repeated. Results were quantified as described by Pfaffl et al. [26]. Specifically developed software solutions tracked samples, standardized processing, and reported analysis results. The procedure for RT‐qPCR analysis is described in more detail by Jonczyk et al. [27].

### 2.5 Detection of SARS‐CoV‐2 specific antibodies

To detect SARS‐CoV‐2‐specific antibodies in serum, two different qualitative and complementary detection methods were used: the electro‐chemiluminescence immunoassay Elecsys Anti‐SARS‐CoV‐2 by Roche Diagnostics (Mannheim, Germany) and the microarray‐based immunoassay SARS‐CoV‐2 ViraChip IgG Test Kit by ViraMed Biotech AG (Planegg, Germany). Elecsys was used to detect IgM and IgG antibodies against the virus’ N antigen. The second test was performed utilizing the ViraChip test kit detecting IgG antibodies against S1, S2, and N virus antigens. Seropositivity was declared when at least one of these antibody types was present.

Elecsys Anti‐SARS‐CoV‐2 was performed with a cobas e411 analyzer by Roche Diagnostics (Mannheim, Germany) using manufacturer's materials and reagents according to manufacturer's instructions directly after serum preparation via centrifugation in serum gel tubes. Apart from sample preparation, analysis using the cobas e411 was fully automated. For the SARS‐CoV‐2 ViraChip IgG test performance, samples were manually transferred into reaction tubes and then diluted by a pipetting robot (rLINE, Sartorius Stedim Biotech, Goettingen, Germany) before manual washing. To increase throughput, the washing of samples was later automated using a Hydroflex Washer by Tecan (Crailsheim, Germany). Test readout was performed with ViraChip Scanner and analyzed with ViraChip Software (Viramed Biotech AG; Planegg, Germany). Antibody detection was set up approximately 4 months after the project started. Sample tracking and analysis report was implemented based on the previously established LIMS and HIS. The detailed procedures for detecting SARS‐CoV‐2‐specific antibodies are described by Jonczyk et al. [27, 28].

### 2.6 Custom LIMS

To account for the specific setup of laboratory and analysis methods, it became necessary to develop a custom LIMS rather than licensing an established LIMS at the project's start. LIMS and HIS development were intentionally split into discrete, stand‐alone software applications. This separation allowed for independent scaling of each application while also facilitating the prospective replacement or integration of custom applications with established LIMS or HIS products.

The LIMS is designed to track every sample, well position, plate, and raw RT‐qPCR and antibody analysis data. The LIMS comprises a document‐based database to store all information. Laboratory routines were aided by software defined processes and direct integration and control of laboratory devices into the LIMS. Users were guided through the necessary manual steps defined by the respective SOP of each analysis by a laboratory user software connected to the database. They were required to confirm steps taken before continuing.

Upon arrival in a laboratory, samples were registered before further processing. The SIDs on labeled specimen tubes are scanned using a handheld scanner and linked to well positions on a plate to accession samples. The LIMS generates a plate definition file containing the SID for each sample loaded into the analysis software that drives the qPCR or ViraChip scanner. Following laboratory analysis, raw data files obtained from laboratory devices are ingested into the LIMS, and each sample was evaluated, resulting in a positive, negative, inconclusive, or invalid result. Following review by senior laboratory scientists, results are confirmed for further processing by the HIS.

### 2.7 Custom HIS

A custom HIS was developed due to the limited time available and desired flexibility of the data acquisition required to enter subject information and link subjects to their respective specimens. The development of the custom HIS was initially focused on utility software used in test kit production and a graphical data entry system deployed at the mobile screening facilities. Printing of the customized adhesive labels was centralized to ensure uniqueness and therefore unambiguousness of provisioned SIDs without the need for sample collection workstations to maintain a constant connection to a centralized server or configuration overhead with the implementation of a local SID provisioning service. This feature allowed subsequent collaborating external laboratories to report lab results in a consistent namespace. Central printing of labels also reduced operating costs instead of printing labels on‐site.

The data entry front‐end system facilitated the acquisition of subject‐specific information such as personal data and state of health and linked the collected specimen to a single subject. Subjects received printouts of the recorded personal data, SID, and legal information during their test appointment. These printouts contained a subject‐specific and encrypted QR code to allow quick recall of subject information during subsequent appointments without requiring a connection to the database system. Acquisition of personal data was initially determined by RKI requirements regarding correct reporting of subjects who tested positive for SARS‐CoV‐2 via PCR [29]. Throughout the project, questions related to ongoing medical research were added to the HIS at different times, with the selection of asked questions adapted based on the target population at the testing location.

The related subject information and clinical data were stored in text‐based files comprising key‐value pairs for the properties of each specimen. This approach ensured compatibility with future additions or alterations to the required data collection. During this initial stage of data entry, the related files were stored locally on encrypted drives and manually backed up. The data entry software ensured complete and conflict‐free storage of files. Files were manually collected from workstation clients for further processing. Upon completion of the laboratory analysis, the result files provided by the LIMS were matched with the respective HIS file in a spreadsheet application and checked twice, followed by manual test result notification.

For PCR tests, subjects received an electronic mail reporting non‐positive results. In case of inconclusive or invalid results, subjects were invited to provide an additional sample. Positive PCR test results were disclosed to medical team members, which then communicated positive results to the subject via phone, emphasizing fast compliance with the required steps mandated by health authorities (e.g., self‐isolation, contact‐tracing). In Germany, a secure digital interface for reporting positive test results was not provided, which required the transmission of positive PCR test results to local health authorities to be performed manually by telefax. A written result was sent electronically to each subject for reporting antibody screening results. Antibody screening results were not required to be disclosed to the authorities.

Advancements towards an automatic workflow of processing lab result reports were made by first automating file transfer from workstation clients to data storage servers with redundant storage and automated backups. Both HIS files and lab results directly obtained from LIMS were periodically parsed into a SQL database. Scripted queries to the database facilitated automation of test result reporting to subjects. Automated reporting of positive PCR test results to local health authorities was set up using a secure online faxing server. The initial manual processes remained valid operation modes throughout the project course to provide fallback methods, for example, in case of connectivity loss or required maintenance. Given collaborations with external laboratories, an import module for structured data was added to the HIS to allow for the import of result files.

### 2.8 Digital infrastructure

**FIGURE 4:**
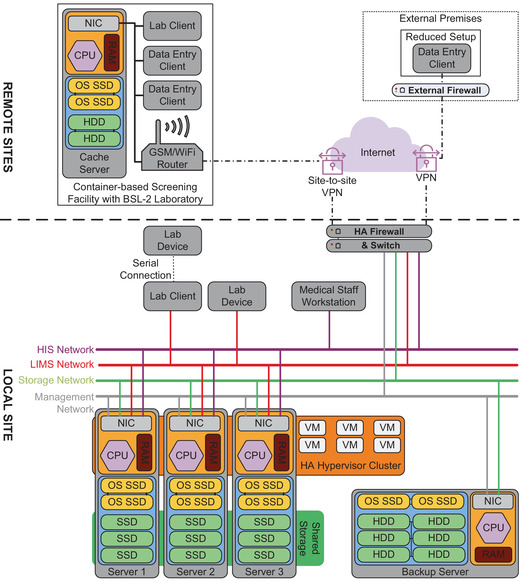
System setup and network diagram. The system setup on campus (“local site”) consisted of three hypervisor servers with shared storage over SSD to establish an HA cluster running the separate VMs for LIMS and HIS database applications. For the OS and storage using HDDs on single servers, a redundant array of independent disks was used. Hypervisor servers were hosted at protected and different locations. A dedicated server handled backups of VMs and data. The separate networks for LIMS, HIS, storage, and server management were governed and protected by an HA firewall. External sites (i.e., container‐based screening facilities with build‐in BSL‐2 laboratories or reduced setups for sample collection) were connected using encrypted VPN connections. CPU, central processing unit; NIC, network interface card; RAM: random access memory

Similar to the provisioning of consumables and safety equipment, the acquisition of technical equipment proved challenging during the initial stage of the SARS‐CoV‐2 pandemic. The project's digital infrastructure relied on stand‐alone encrypted clients and manual data backup and transfer tasks in the first few weeks. Once the availability of hardware components improved, the digital infrastructure was scaled to a reliable server setup. Figure 4 depicts the detailed setup of servers and the network infrastructure.

LIMS and HIS databases were deployed using virtual machines (VM) on centralized servers. A set of three hypervisor servers with shared storage was set up to provide a high availability (HA) environment featuring live migration of VMs to ensure uninterrupted operation even during server maintenance. Additional servers were deployed for regular backups. Servers were distributed among different buildings and backed by uninterruptible power supplies (UPS) to eliminate any single point of failure or data loss in case of power outages. Physical access to servers was limited to system administrators. Staff members only received access to the deployed clients upon completion of a technical briefing and data regulations and safety training. Authentication of users was governed by domain‐based user administration.

An HA firewall protected internal networks that allowed granular access from external authenticated devices and users using virtual private network (VPN) connections. Unidirectional flow of subject‐related data from remote locations (i.e., mobile screening facilities) was established. All client‐to‐server communication was encrypted. Container‐based screening facilities included a locally placed encrypted server with redundant storage for temporary caching of files and mirroring of domain information. This ensured stable operation in the cases of poor internet availability on‐site.

### 2.9 Quality management and regulatory affairs

The laboratory operation was subject to extensive quality management and compliance with applicable policies and guidelines to establish and maintain the required quality, safety, and performance in the use of diagnostics, and to ensure the reliability of the results obtained with them. Certification of the laboratory operation was achieved in order to be able to report the obtained results to subjects and local health authorities. For this purpose, a quality management system in accordance with the “RiLiBÄK” directive (“Richtlinie der Bundesärztekammer zur Qualitätssicherung laboratoriumsmedizinischer Untersuchungen”; accompanied by Labor Limbach, Lehrte, Germany) was established. This directive describes basic requirements (e.g., appropriate spatial conditions, provisioning of equipment documentation, written preanalytical measures) for quality assurance.

The safety concept and the prerequisites for operation were precisely documented in SOPs, risk assessments and hygiene plans, and checked and confirmed by the responsible occupational safety department (i.e., biological safety officer) and the trade supervisory office. Operating instructions were prepared in accordance with the RiLiBÄK specifications for devices, analysis systems, and procedures and released after external review. The instructions were regularly updated and were accessible both digitally and at the workplace for authorized personnel. Before using the respective laboratory methods, especially after method modification, they were verified in at least three independent test runs with samples whose test results were known. The verification of RNA isolation and PCR analysis was performed using 30 samples, the test procedures of the antibody module were each verified with 260 samples of different origin. To assess the quality of the PCR analysis, laboratory operations were tested by an external quality assurance service program for the detection of the virus genom (provided by INSTAND; Düsseldorf, Germany).

To ensure reliable operation over time, the guidelines provide for regular maintenance and calibration of the equipment in accordance with the manufacturer's specifications. Quality control for PCR analysis included an extraction control in the form of detecting human RNA for each specimen. Positive and negative controls were added to each plate and verified during PCR analysis. For quality control of the antibody detection using the Roche automated electrochemiluminescence immunoassay, control samples provided by the manufacturer were analyzed twice daily. For the antibody detection using the microarray‐based immunoassay SARS‐CoV‐2 ViraChip IgG Test Kit, control samples from the manufacturer with known results were integrated on each analysis plate. In addition, internal controls (serum control, IgG control, negative control, and calibrators) are located in each well by default to perform a functional check for each analyzed sample.

A separate data protection concept for handling the collected subject data was drafted and submitted to the appropriate data protection officer for review. It complies with Article 5 of the General Data Protection Regulation (GDPR) and the data protection principles and other data protection requirements set out therein. An ethics vote of the responsible medical association (Lower Saxony, Germany) and Institutional Review Board approved the evaluation of the collected subject data in a pseudonymized form in the context of medical studies (No. Bo/30/2020; Bo/31/2020; Bo/32/2020; 9085_BO_S_2020).

## 3 RESULTS AND DISCUSSION

Less than 4 weeks after the first imposed lockdown in Germany, and despite many issues regarding the provision of required materials (e.g., consumables, technical devices, and safety equipment), a first mobile container‐based testing station commenced operation in April 2020 [30]. In addition to subject admission and sample collection, RNA isolation could be performed in this station due to the build‐in laboratory meeting BSL‐2 requirements. Samples were sent to BSL‐1 laboratories for PCR analysis after RNA isolation. Shortages in supply chains were overcome by provisioning custom test kits, establishing in‐house logistics for materials and samples, and developing both a LIMS and HIS tailored to the sample collection and laboratory analysis processes. Initially, handling of samples and reporting analysis results to subjects and local authorities were not automated. The operation of the screening facilities and diagnostic laboratories following this implementation was approved by the responsible authorities. A concluding data protection clearance was issued based on the implemented data handling. The quality of PCR diagnostics was verified by successful participation in an external quality assurance service program. Therefore, this minimum‐viable product already met the appropriate safety requirements and followed extensive quality management but required substantial daily human intervention.

The container‐based setup with a built‐in BSL‐2 laboratory enabled high throughput of sample acquisition and processing with minimum requirements posed to the installation site. The shipping containers allowed easy relocation and required a footprint of 33 m^2^. A three‐phase power supply was required to operate the test station, which a mobile generator can also provide. The water needed for the operation was provided through water tanks. Subject and sample data was able to be transmitted to a central server via encrypted mobile storage media if no active internet connection was available on‐site. These design considerations allow for a flexible setup even in rural areas. However, the correct and safe implementation of PCR and antibody detection tests is limited to trained personnel, which might become challenging in some areas. Here, the decentralized collection of samples and subsequent transport to a central laboratory presented a common approach that can reduce the demand for biotechnology professionals. The presented setups were used for decentralized sample collection feeding into the container‐based BSL‐2 laboratories for RNA isolation and subsequent transport to campus laboratories for PCR analysis. The lack of centralized BSL‐2 laboratories still created a high need for personnel to operate each laboratory station.

The setup was constantly improved during the course of the project and, in particular, extended to include automation steps regarding laboratory analysis and reporting of results. Due to the forward compatibility considered from the beginning and the iterative improvement of processes, this approach allowed the setup to be launched quickly and scaled at a fast pace. Over time, subjects also became familiar with the PCR testing procedure and the collection of personal data and clinical parameters was significantly accelerated. Here, the personal QR codes, in particular, played a major role, which reduced the initial average of 8 min for capturing subject data and taking the swab to approximately 4 min. With these improvements, at a single mobile container‐based screening facility over 220 tests were collected in 8 h. Swabs taken before noon in a container‐based screening facility close to the university's BSL‐1 laboratories used for PCR analysis could be evaluated the same day. Most other samples could be analyzed within 36 h.

The devised screening facilities and diagnostic laboratory were successfully used in the context of two medical studies. Table 2 displays the number of screening facilities, subjects, and samples associated with each study. As part of the first study, employees of various educational institutions, companies, and nursing homes were offered voluntary PCR and antibody testing close to their respective workplaces or places of living (Cohort 1) [27, 28]. The second study focused on pupils, at least one of their close relatives, and faculty members of two schools (Cohort 2) [31, 32].

**TABLE 2:** Number of deployed test stations, participating test subjects, and tests analyzed for each of the different cohorts (Cohort 1: Employees of different educational institutions, companies, and nursing homes; Cohort 2: Pupils, at least one of their close relatives, and faculty members of two schools)

		Cohort 1	Cohort 2	Total
Screening facilities	Container‐based setup with BSL‐2 laboratory (PCR)	2	1	3
	Reduced setup (PCR)	10	1	11
	Reduced setup (bloodsampling)	6	1	7
Subjects	Participated in PCR testing	4817	2463	7280
	Tested positive for acute SARS‐CoV‐2 infection by PCR	51	2	53
	Participated in antibody detection testing	1883	2179	4062
Samples	Samples for PCR analysis	26,908	6405	33,313
	Samples for antibody detection analysis	2696	4167	6863

**FIGURE 5:**
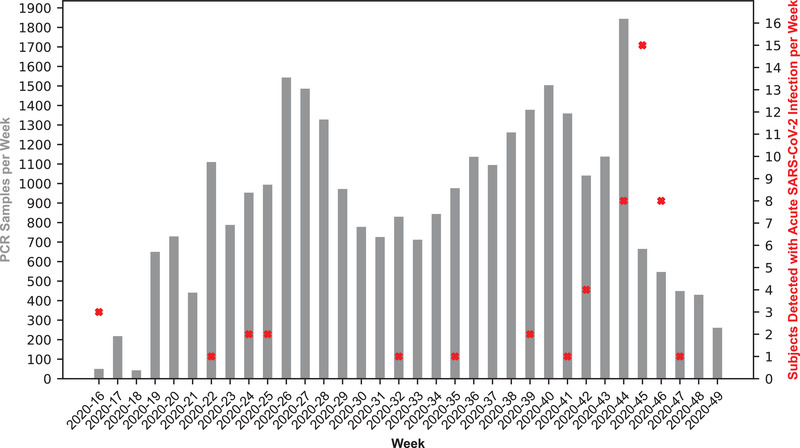
Number of PCR tests and subjects tested positive for SARS‐CoV‐2 per week. PCR tests and subjects with a PCR confirmed acute SARS‐CoV‐2 infection recorded at a mobile screening facility are displayed per week for the time period between April 2020 and November 2020

Figure 5 displays the number of recorded PCR tests per week and the number of total subjects with PCR confirmed SARS‐CoV‐2 infection between April 2020 and November 2020. This period represented the busiest phase of the project, characterized by the simultaneous operation of three container‐based screening facilities and ten additional test sites relying on the reduced setup. At the beginning of 2021, rapid antigen tests for detecting SARS‐CoV‐2 became available and have been provided by many employers for their employees to test themselves. In addition, the approval of the COVID‐19 vaccines occurred in Germany at the beginning of 2021, followed by administration of vaccine shots soon after. These vaccines can reduce the risk of contracting and spreading a SARS‐CoV‐2 infection, while also possibly mitigating the course of disease [33, 34]. In light of these developments, operations scaled down to two container‐based screening facilities and three reduced setups focusing on educational institutions. PCR screening and antibody detection continued until June 2021.

More than 33,000 PCR tests and close to 7000 antibody detection tests were performed. Over 7200 subjects participated in the PCR test offer, and more than 4000 subjects participated in antibody detection tests. Subjects accepted the offer for PCR testing with varying frequencies. In Cohort 1, the number of PCR tests performed ranged widely between one and 46 tests per subject, with a median of three tests. About 31% of the subjects underwent PCR testing only once during the several months of the project period. For population screening using PCR tests to be an effective measure, it must be coupled with strict precautionary measures (e.g., hygiene concept) and a high participation rate of subjects at risk [35]. Compared to rapid antigen tests, PCR tests provide a better sensitivity with acceptable turnaround times and limit the amount of false‐positive and false‐negative results, which can have considerable health, economic, and social impacts [36, 37]. The higher costs associated with using PCR tests for screening compared to rapid antigen tests need to be traded off against the costs and damage caused by a potential SARS‐CoV‐2 infection [38]. Limiting PCR screening by targeting populations with an increased risk of infection based on their occupational situation or medical condition presents one possible approach to reduce costs while still detecting even asymptomatic and presymptomatic subjects before wide spread of infections. Screening projects such as the one described here depend on a rapid definition of target populations by official bodies in order to be particularly effective.

Overall, 53 subjects with acute SARS‐CoV‐2 infection were detected by PCR analysis (total prevalence of 0.7%). After a confirmed positive PCR test in one nursing home, thanks to the quick deployment of a mobile test station, residents and staff members were tested repeatedly, documenting a localized outbreak in late October of 2020 (see Figure 5; weeks 2020–45 and 2020–46). This outbreak fell within a period of high incidence, in which the provision of additional testing capacity can play an important role in easing the burden on public health departments [39]. The medical team's direct care and information sharing allowed important actions such as isolation and contact tracing to be initiated quickly. It was observed that subjects who tested positive for SARS‐CoV‐2 within the targeted population groups showed overall good compliance with measures regarding self‐isolation and contact‐tracing limiting infection spreading.

The antibody screening tests revealed 58 not‐vaccinated subjects in Cohort 1 that never had a positive PCR test taken but displayed seroconversion, indicating a subclinical infection with SARS‐CoV‐2 [27]. A relatively high rate of subclinical SARS‐CoV‐2 infections may contribute to the spread of SARS‐CoV‐2, suggesting that in addition to other intervention strategies, systematic screening of asymptomatic persons by PCR testing may significantly enable better pandemic control.

PCR screening of pupils and faculty members of two schools (Cohort 2) showed a low infection rate among these subjects (see Table 2). This indicates that in‐person learning can occur safely if extensive protective measures are in place and the incidence in the general population remains moderate [31]. The acquisition of additional parameters beyond the clinically relevant data with the help of the implemented data entry software also revealed that age and parental level of education influence COVID‐19 vaccine hesitancy. This especially concerns children under the age of 16 and students whose parents had lower education levels, which showed significantly higher vaccine hesitancy. Identifying populations that exhibit greater reluctance to implement key pandemic control measures is important for designing public information campaigns [32].

The setup and operation of mobile SARS‐CoV‐2 screening facilities and diagnostic laboratories represents a very challenging project regarding material and personnel expenses. Considering all expense factors (e.g., material, laboratory, and personnel costs), the total cost per PCR test within this project was approximately 60 €, divided equally between sample collection (including transport) and lab costs. Despite the added overhead of sample collection on‐site, performing a PCR test within this project was only slightly more expensive than the billable cost (i.e., 52.50 €) of preventive PCR testing of hospitalized subjects in Germany [40]. Due to the legal regulations in Germany, the costs incurred per PCR test within the project's framework could not be settled via a health insurance [41].

Within this project's scope, setups and operating procedures for outdoor and indoor screening facilities were developed. These mobile setups, suitable for a flexible choice of location, made it possible to bring the test offer close to the target populations’ workplace or place of living. Furthermore, the container‐based screening facilities included BSL‐2 laboratories providing additional capacities to process the potentially infectious samples. Table S1 provides an overview of testing strategies developed by other groups in response to the SARS‐CoV‐2 pandemic. A direct comparison between the different setups is complicated due to the many factors to consider (e.g., testing modalities, location, target population size, test frequency, test acceptance, and operation costs). Most publications also only reported on some key characteristics. However, it is clear that flexible testing approaches are needed to provide sufficient and equal access to testing capacity for all members of society. Lessons learned in this project are that close cooperation with established diagnostic laboratories can significantly accelerate the setup process. Secure digital recording and transfer of patient and laboratory data are central to ensuring rapid and reliable communication of results. Automating laboratory processes and subject data handling should be aimed at an early stage to reduce the required staff considerably. Based on the achieved sample throughput, quality of the diagnostic laboratory, running costs, and medical findings, the presented concept can be a valuable complement to testing strategies, especially where a fast response is required or access is otherwise restricted.

Much of the testing shortfall, especially early in the pandemic, can be traced to a shortage in critical supply chains, personnel required, expertise, or instrumentation necessary to collect samples and perform PCR analysis. University‐based research laboratories can provide much‐needed assistance to increase PCR testing capacity. However, adjusting legal frameworks (e.g., preparation of disinfectant or ease of transport regulations) can become necessary to allow operation under the difficult conditions imposed by a pandemic. Improving the digital infrastructure in public health can lead to the removal of barriers to enable simplified connection of university‐based research laboratories to the health care system in times of crisis. In addition, government funding often becomes necessary for university‐based research laboratories to account for the extensive costs.

## 4 CONCLUSION

Population screening regardless of the presence of COVID‐19 related symptoms using PCR tests represents a valuable method to limit the spread of SARS‐CoV‐2. Targeted screening of subjects in medical, educational, or system‐critical professions and subjects at high risk of severe disease progression can help to maintain critical infrastructure and reduce mortality rates.

This report describes the implementation, continuous optimization, and operation of mobile screening facilities and diagnostic laboratories by non‐medical university research institutions during the first 15 months of the SARS‐CoV‐2 pandemic.

In line with implementations and shared experiences from other groups, it is clear that university‐based research laboratories represent an important resource when facing a pandemic scenario. The authors recommend government entities to financially support universities with the capability of adding to the testing capacity and provide the required infrastructure, legal framework, and health policy guidelines to enable the university‐based operation of diagnostic laboratories to effectively combat pandemic scenarios. This report can assist others in quickly establishing similar setups valuable for pandemic response.

## CONFLICT OF INTEREST

The authors have declared no conflicts of interest.

## ETHICS STATEMENT

An ethics vote of the responsible medical association (Lower Saxony, Germany) and Institutional Review Board approved the evaluation of the collected subject data in a pseudonymized form in the context of medical studies (No. Bo/30/2020; Bo/31/2020; Bo/32/2020; 9085_BO_S_2020).

## INFORMED CONSENT

Informed consent was obtained as part of the digital registration process at the mobile testing sites.

## Supporting information

SUPPORTING MATERIAL

## Data Availability

The data that support the findings of this study are available from the corresponding author upon reasonable request.
