# Supplementary material for: Mobile SARS‑CoV‑2 screening facilities for rapid deployment and university‐based diagnostic laboratory
Source: Eng Life Sci. 2023 Jan 3;23(2):2200026. doi: 10.1002/elsc.202200026 (PMC9893752; doi:10.1002/elsc.202200026)
Supplement: Supplementary file 1 — SUPPORTING MATERIAL [file ELSC-23-2200026-s001.pdf]

Table S1: Characteristics of SARS-CoV-2 testing facilities.

Comparison item	this work	Stemler et al. [6]	Shah et al. [7]	Mark et al. [8]	Muhi et al. [10]	Chang et al. [12]
**Country**	Germany	Germany	USA	Scotland	Australia	USA
**Testing modality**	Outdoor & indoor walk-through sites with mobile setups	Mobile testing site	Drive-through center	Home visiting testing	Outdoor walk-through site (fixed location)	
**Target population**	University and school staff members, pupils (and their relatives), nursing home staff members & residents, workers in system critical professions	Nursing home staff members and visitors	n/a	n/a	University members	University members
**Project period**	Apr'20 - Jun'21	Oct'20 - Dez'20	Mar'20 - Apr'20	Feb'20	Nov'20 - Dez'20	Apr'20 - May'21
**Sampling location**	Container-based setups: university, school, company grounds<br>Reduced setups: schools, nursing homes, company grounds	Nursing homes (4)	Hospital grounds	At home visits	University campus (car park)	University campus
**Laboratory location**	BSL-2 laboratory included in container-based setups for RNA isolation<br>RT-PCR at central laboratory in university	Central laboratory	Central labortory	Central labortory	BSL-2 laboratory at sample location for RT-PCR at central laboratory	Central laboratory
**Testing facilities deployed**	3x Container-based setups with BSL-2 laboratory<br>11x Reduced setup for sample collection	1x mobile testing vehicle	1x Drive-through center with 4 tent-covered lanes and 50m waiting area	1x mobile testing vehicle	1x Container- & tent-based setup	n/a
**Spatial requirements**	Container-based setup: 2x Shipping Container (6m x 2.6m)	1x mobile testing vehicle	1x Drive-through center with 4 tent-covered lanes and 50m waiting area	1x mobile testing vehicle	1x Shipping Container (6m x 2.6m),<br>2-3x tents (6m x 3m),<br>1x tent (3m x 3m)	n/a
**Power supply**	On-site three phase mains power or power generator	n/a	n/a	n/a	Dual 10A power supply	n/a
**Water supply**	Water tanks	n/a	n/a	n/a	Public water supply	n/a
**Number of participants**	7,280	n/a	n/a	79	415	
**Total tests performed**	33,313	1,587	approx. 7,700	79	n/a	n/a
**Test methods**	RT-PCR	RT-PCR for symptomatic subjects or SARS-CoV-2 RNA positive pools	RT-PCR	n/a	RT-PCR & point-of-care-tests	
**Maximum sampling throughput**	approx. 220 in one 8h shift in one container-based setup	approx. 70 per day	approx. 400 per day	11 per day	n/a	approx. 1600 per day
**Turnaround time**	Samples taken before noon: <12h<br>other samples: <36h	same-day result notification	< 24h	n/a	RT-PCR: 24h<br>point-of-care-tests: 32-75min	~25h (STD: 11h)
**Result notification**	E-Mail;<br>phone call if PCR positive	text message to phone;<br>phone call if PCR positive	Online service and messages to phone; phone call if PCR positive	n/a	text message to phone;<br>pos. PCR results reported to clinician	E-Mail
**Staff**	per shift (8h)<br>Container-based setup: 4 (sampling: 2; lab: 2)<br>Reduced setup: 1<br>Central laboratory (PCR analysis): 2<br><br>overall<br>>150 staff members	n/a	Sampling: 20 - 24 + supervisor;<br>Phone line: 60 - 70;<br>Additional staff for clinical & organizational supervision	Mobile testing vehicle: 3<br>Central labortory: n/a	n/a	n/a
**Duration of sampling (average)**	5 min	n/a	n/a	30 min + <1 h drive	n/a	n/a
**Cost per test**	approx. 60€	n/a	n/a	£55 (only staff costs considered)	n/a	n/a
